# Imaging-Based Risk Stratification of IPMN Using a Structured Imaging Score: A Retrospective Proof-of-Concept Study

**DOI:** 10.3390/curroncol33070383

**Published:** 2026-06-24

**Authors:** Stefano Fusco, Hannes F. Digomann, Sabrina Groß, Nisar Peter Malek, Eckhart Fröhlich, Tatjana Hoffmann

**Affiliations:** 1 Section of Gastroenterology, Oncology and Palliative Care, Department of Internal Medicine 1, Medical Campus Lake Constance, 88048 Friedrichshafen, Germany; 2 Department of Internal Medicine I, Marienhospital Stuttgart, 70199 Stuttgart, Germany; hannes.digomann@vinzenz.de; 3 Section of Gastroenterology, Gastrointestinal Oncology, Hepatology, Infectiology and Geriatrics, Department of Internal Medicine I, University Hospital of Tübingen, 72076 Tübingen, Germany; sabrina.gross@med.uni-tuebingen.de (S.G.); nisar.malek@med.uni-tuebingen.de (N.P.M.); eckhart.froehlich@gmx.de (E.F.); tatjana.hoffmann@med.uni-tuebingen.de (T.H.)

**Keywords:** IPMN, cystic pancreas lesion, Fukuoka criteria, Tübingen Dignity Score, TDS

## Abstract

Background/Objectives: Accurate risk stratification of intraductal papillary mucinous neoplasms (IPMNs) remains clinically challenging. This study evaluates a structured imaging-based scoring approach for IPMN risk stratification, referred to as the Tübingen Dignity Score (TDS), and compares its diagnostic performance with established methods. Methods: In this retrospective study, imaging findings from patients with suspected IPMN were analyzed using MRI, CT, and ultrasound. The TDS was applied as an imaging-based scoring system. Diagnostic performance was assessed in a histopathological subset and compared with MRI-based assessment and Fukuoka criteria. Results: MRI showed high sensitivity (94.4%) but limited specificity (57.1%). Fukuoka criteria showed high sensitivity (100%) and high specificity (91.3%) in this cohort, although with a lower positive predictive value. In contrast, the TDS showed high specificity (100%) and positive predictive value, but lower sensitivity (40%), reflecting a different diagnostic profile. These findings indicate a trade-off between sensitivity and specificity across the evaluated approaches. However, the limited number of malignant cases limits the robustness and generalizability of these estimates. Conclusions: The TDS may serve as a complementary, imaging-based tool within a multimodal diagnostic framework for IPMN. Its potential value lies in supporting clinical decision-making in selected cases, particularly where established criteria yield inconclusive results. Given the limited sample size, retrospective single-center design, and exploratory nature of this study, external prospective multicenter validation is required before routine clinical application can be recommended.

## Simple Summary

Intraductal papillary mucinous neoplasms (IPMNs) are cystic lesions of the pancreas that can remain harmless but may also develop into pancreatic cancer. Distinguishing low-risk from high-risk lesions before surgery is challenging and often leads to uncertainty in clinical decision-making. In this study, we evaluated a structured imaging-based scoring approach for IPMN risk stratification, referred to in this study as the Tübingen Dignity Score (TDS). The score combines guideline-based imaging features and can be applied to MRI, CT, and ultrasound examinations. Our results suggest that the TDS performs best when applied to MRI and shows agreement with histopathological findings in this cohort, although based on a limited sample size. MRI-based TDS identified patterns consistent with low-risk and high-risk lesions, while CT and ultrasound showed moderate agreement with MRI. The TDS may support clinical decision-making regarding surgical versus conservative management, particularly in cases where risk assessment remains inconclusive.

## 1. Introduction

Intraductal papillary mucinous neoplasms (IPMNs) of the pancreas are mucin-producing cystic epithelial lesions and are among the most commonly detected pancreatic cystic neoplasms. Their clinical relevance lies in their potential to progress to pancreatic ductal adenocarcinoma (PDAC), a malignancy with a poor prognosis when detected at an advanced stage [1,2,3,4,5,6]. The incidence of IPMNs has increased significantly over the last decade due to widespread use of high-resolution imaging modalities, particularly MRI and CT, which frequently identify pancreatic cysts incidentally [7,8]. Despite their increased detection, only a subset of IPMNs develop high-grade dysplasia or invasive carcinoma, while the majority remain indolent, making accurate preoperative risk stratification essential to avoid unnecessary surgery and its associated morbidity [9].

IPMNs are classified anatomically into main duct (MD), branch duct (BD), or mixed types, and histologically into gastric, intestinal, pancreatobiliary, or oncocytic subtypes, each associated with distinct malignant potential [4,10]. Imaging features such as main pancreatic duct dilation, enhancing mural nodules, cyst wall thickening, and rapid cyst growth have been associated with higher malignancy risk, according to the Fukuoka criteria [4,11,12,13,14]. Serum biomarkers, including CA19-9 and CEA, can provide additional guidance, though their specificity is limited [15,16]. BD-IPMNs are more common but generally carry lower malignant potential than MD-IPMNs, complicating management decisions [3,6].

Current international consensus guidelines, including the Fukuoka and Kyoto guidelines, recommend surgical resection for lesions with high-risk stigmata while suggesting surveillance for low-risk lesions [3,4,6]. However, several studies have demonstrated that a proportion of lesions deemed high-risk are ultimately low-grade on histopathology, whereas some malignant lesions are missed preoperatively [8,9,12,15,17,18,19]. This highlights the limitations of existing criteria and underscores the need for improved preoperative risk stratification to balance intervention against overtreatment [7,8,17].

Recent efforts have focused on predictive models integrating clinical, imaging, and molecular data. Nomograms based on CT or MRI findings have demonstrated improved discrimination for malignant IPMNs compared with guideline criteria alone [7,8,11]. Molecular profiling of cyst fluid, including mutations in KRAS, GNAS, RNF43, and TP53, can further refine risk assessment [20,21,22]. Longitudinal surveillance incorporating cyst growth and evolving imaging features enhances prediction of malignant transformation beyond static assessment [19,21,22].

Despite these advances, no universally accepted scoring system currently integrates multimodal data to reliably predict IPMN malignancy risk [6,16,23].

To address this gap, an exploratory structured imaging-based scoring approach, referred to as the Tübingen Dignity Score (TDS), was developed as a composite system to differentiate low-grade dysplasia from high-grade dysplasia or invasive carcinoma.

This study addresses the following key questions:

- MRI-TDS vs. histopathology: How accurately does the MRI-based TDS, as a non-invasive imaging-based assessment, reflect definitive histopathological diagnoses?
- CT-TDS and ultrasound-TDS vs. MRI-TDS: How closely do CT- and ultrasound-derived TDSs correlate with MRI-TDS to assess alternative imaging reliability?

By addressing these questions, the present study aims to evaluate the TDS as a pragmatic imaging-based risk stratification tool and to assess its consistency across imaging modalities in comparison with histopathological findings.

## 2. Materials and Methods

### 2.1. Study Design and Patient Cohort

The retrospective evaluation was performed at the Medical Clinic I of the University of Tübingen after Institutional Review Board (IRB) approval (project number: 407/2017BO2). Patients with a diagnosis of intraductal papillary mucinous neoplasm (IPMN) between 2005 and 2018 were eligible for inclusion if IPMN was suspected on at least one imaging examination and/or confirmed by histopathological evaluation of the pancreas. Patients were included if they had suspected IPMN based on imaging findings and underwent at least one cross-sectional imaging examination (MRI, CT, EUS or transabdominal ultrasound) during the study period. Patients who underwent surgical resection were included in the histopathological subset. Because the study aimed to evaluate imaging-based preoperative risk stratification in a real-world clinical setting, patients with postoperative non-IPMN diagnoses were initially retained within the surgically resected histopathological cohort. Their inclusion reflects the clinical challenge of differentiating suspected IPMN from alternative pancreatic cystic or malignant lesions during preoperative imaging assessment. However, these cases were excluded from analyses specifically evaluating IPMN-related diagnostic performance in order to ensure a consistent reference standard focused on histopathologically confirmed IPMN.

The ICD-O-3 code (International Classification of Diseases for Oncology, 3rd Edition) was used to classify neoplastic entities, providing standardized international coding for tumor morphology and behavior. Patients in whom the (suspected) diagnosis of IPMN was not confirmed in any imaging or histopathological examination and whose examinations had an inter-examination interval > 1 year were excluded.

**Figure 1:**
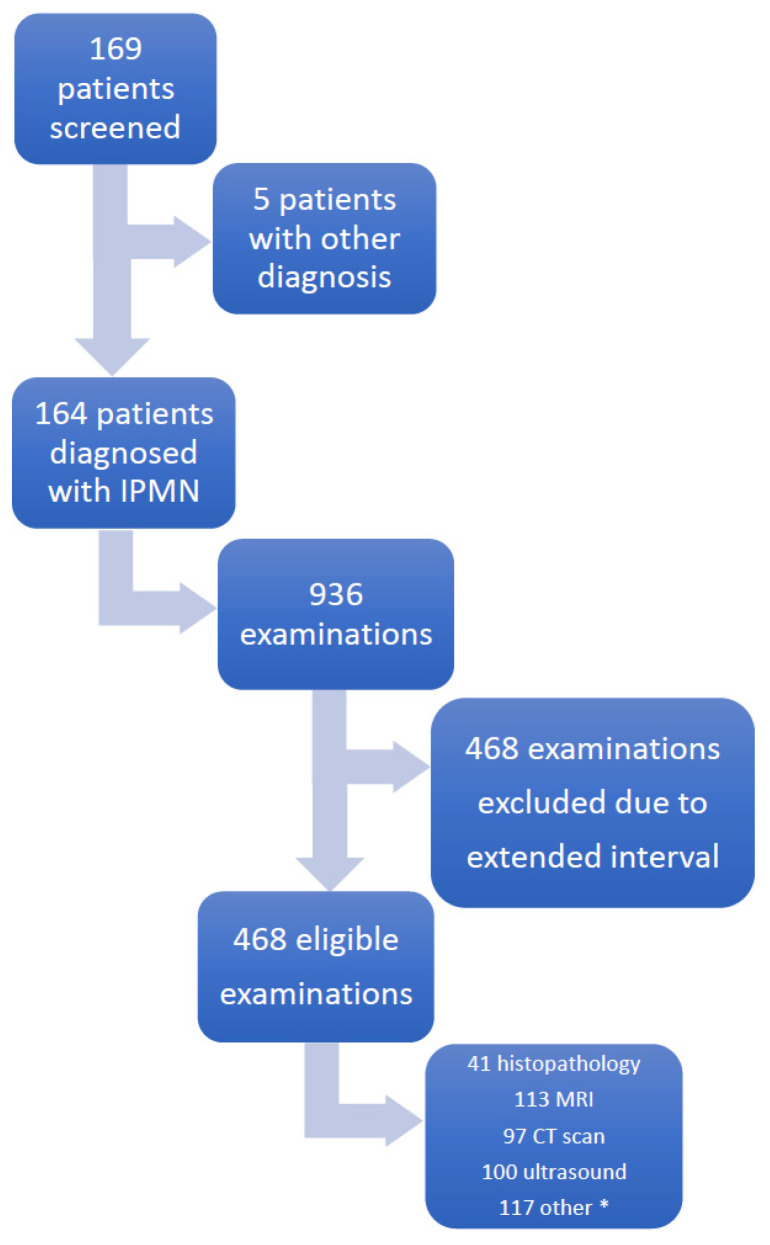
Study flow diagram. * Other: ERCP, 3D-US, EUS.

The study selection process and patient flow are summarized in Figure 1. A total of 169 patients with suspected IPMN were initially identified. After exclusion of five patients due to alternative diagnoses, 164 patients constituted the final study population. Within this cohort, 936 imaging and histopathological examinations were reviewed. To ensure methodological consistency and temporal comparability, examinations with an interval greater than one year between the individual examination and the reference examination were excluded, resulting in 468 eligible examinations for analysis.

### 2.2. Imaging Analysis

All cyst, pancreatic duct, and lymph node data were recorded, as well as the examiner’s diagnosis:

- The greatest extent of the cyst in millimeters, detection or exclusion of mural nodules, septa, or mural thickening, and perfusion status.
- The ductal extent of the main pancreatic duct in millimeters, and three groups were formed:
  - <5 mm or not dilated;
  - 5–9.9 mm or dilated without further size information;
  - ≥10 mm.
- Presence or exclusion of caliber mismatch in the pancreatic duct.
- Lymph node enlargement or lack thereof.

MRI served as the primary reference modality for imaging-based comparisons because of its established sensitivity for IPMN assessment. The same imaging parameters were recorded for MRI, CT, and ultrasound examinations. Blood flow was additionally assessed using contrast-enhanced imaging or color Doppler ultrasound, where available.

CT examinations frequently represented the initial imaging modality, particularly in patients referred from external institutions or evaluated outside specialized pancreatic centers. Dedicated MRI examinations were often performed subsequently for further characterization of suspected pancreatic cystic lesions.

### 2.3. Histopathological Analysis

Pathology served as the gold standard for the examinations in terms of diagnosis and malignancy risk. The following parameters were recorded for each pathology examination: Date of examination, diagnosis, IPMN proven, risk of malignancy, histological type, pancreatic duct (size, widening, caliber leaps), cysts (localization, number, size, reference, mural nodules, septs, thickening of the wall) and lymphnodes. Histopathology served as the reference standard for diagnostic performance analyses, whereas MRI was used as the reference modality exclusively for intermodality comparisons.

### 2.4. Tübingen Dignity Score (TDS)

An exploratory risk stratification score (referred to throughout this manuscript as the Tübingen Dignity Score [TDS]) was originally proposed by Digomann ([24] Digomann 2021, doctoral thesis, University of Tübingen, Germany) for standardization of imaging findings. The TDS was based on the parameters of the guidelines with different weighting. The weighting of the individual TDS parameters was developed as a heuristic clinical scoring approach within the framework of the underlying doctoral thesis. Weight assignment was based on the perceived relative clinical relevance of imaging findings reported in international guideline recommendations and prior literature regarding IPMN-associated malignancy risk.

Imaging features considered to represent low-risk morphological changes (e.g., limited ductal alterations) received lower scores, whereas findings associated with increasing concern for malignant transformation, such as mural nodules, received substantially higher weighting. The markedly higher weighting assigned to mural nodules reflected their established association with high-grade dysplasia and invasive carcinoma in guideline-based IPMN risk stratification concepts.

The weighting system was not derived from formal multivariable statistical modeling or odds-ratio-based regression analysis and should therefore be interpreted as an exploratory, clinically motivated scoring framework requiring prospective validation and potential future refinement. The threshold of 25 points was chosen pragmatically within the original scoring concept to ensure that imaging findings considered highly suggestive of malignancy (e.g., mural nodules ≥ 5 mm or MPD ≥ 10 mm) directly resulted in high-risk classification.

### 2.5. Comparative Classification Using Fukuoka Criteria

The International Consensus Guidelines and the European Guidelines divide IPMN or cystic pancreatic lesions into three risk areas:

1. Into an absolute indication for surgery, the “high-risk” area in which the patient should be operated.
2. Into a relative surgical indication, the “worrisome” area in which surgery should be considered, depending on the findings and the guidelines.
3. Into a “worthy of observation” or “worth watching” range, where it is recommended that the lesion, at timed intervals, be regularly monitored.

The TDS classification and weighting of findings are shown in Table 1.

For comparative diagnostic analyses, the revised Fukuoka criteria were retrospectively applied to the same subset of patients with histopathologically confirmed IPMN (n = 25). This approach ensured methodological consistency across all evaluated diagnostic strategies and enabled direct comparison between MRI assessment, the Tübingen Dignity Score (TDS), and Fukuoka-based risk stratification. Patients were categorized according to established Fukuoka risk groups based on the presence of high-risk stigmata and worrisome features. Diagnostic performance metrics were subsequently calculated using histopathological findings as the reference standard.

### 2.6. Statistical Analysis

The determination of sensitivity and comparison of the TDS was performed with cross-tabulations, and the degree of agreement was calculated with Cohen’s Kappa. The interpretation of the value was done according to the ranges proposed by Landis and Koch [25]. Results were considered significant at *p* ≤ 0.05. Descriptive statistics were expressed as mean ± SD or median (IQR). Categorical variables were compared using the chi-square test or Fisher’s exact test, as appropriate. Continuous variables were assessed for normality and are presented as mean ± standard deviation or median (interquartile range), depending on distribution. Group comparisons for continuous variables were performed using Student’s *t*-test for normally distributed data and the Mann–Whitney U test for non-normally distributed data. Statistical significance was set at *p* < 0.05. Analyses were performed using SPSS version 29.0 (IBM Corp., Armonk, NY, USA) and R version 4.3.0 (R Foundation for Statistical Computing, Vienna, Austria).

## 3. Results

### 3.1. Patient Cohort and Available Examinations

The final analysis included 468 eligible examinations, consisting of 41 histopathological examinations, 113 MRI examinations, 97 CT examinations, and 100 transabdominal ultrasound (US) examinations. This distribution reflects routine clinical imaging practice over the long observation period. The overlap between available imaging modalities and histopathological examinations is summarized in Table 2.

### 3.2. Histopathology

Histopathological examination was available in 41 cases and served as the reference standard for diagnostic performance analyses. IPMN was confirmed in 61% (25/41) of cases, whereas the remaining 39% (16/41) comprised alternative postoperative diagnoses, including pancreatic adenocarcinoma with coexisting IPMN (n = 10), pancreatic adenocarcinoma without coexisting IPMN (n = 4), cystadenoma (n = 1), and pancreatic cyst (n = 1). These non-IPMN cases were excluded from IPMN-specific analyses to ensure a consistent reference standard focused on histopathologically confirmed IPMN. The IPMN-positive subgroup comprised 40% female (10/25) and 60% male (15/25) patients, with a mean age of 66.8 ± 8.8 years, consistent with the typical demographic distribution reported for IPMN (Table 3). Within the histopathologically confirmed IPMN subgroup (n = 25), malignant transformation was identified in 2 cases, while 23 lesions were classified as benign.

Table 2 summarizes the overlap between histopathological examinations and imaging modalities, as well as between MRI and other imaging techniques within the retrospective cohort. A total of 25 cases had both histopathology and MRI available, while overlap with CT and ultrasound was more frequent, reflecting the broader use of these modalities during the study period. The second row illustrates the overlap between MRI and CT, ultrasound, and 3D ultrasound examinations, thereby demonstrating the extent of multimodal imaging within the cohort.

### 3.3. Distribution of the Tübingen Dignity Score (TDS) Across Imaging Modalities

#### 3.3.1. MRI

The TDS could be calculated in 111 of 113 MRI examinations, reflecting high completeness of imaging data. The majority of MRI examinations were classified as worth watching (64.9%), followed by worrisome (32.4%) and high-risk (2.7%) categories, indicating a predominance of low-to-intermediate-risk imaging features. As shown in Table 4, all lesions classified as high risk by the TDS corresponded to malignant histopathology (100%, 2/2; 95% CI: 15.8–100), although this finding is based on a very small sample size and should therefore be interpreted with caution, whereas all lesions in the worth watching category were histologically benign (100%, 7/7). Lesions classified as worrisome showed mixed results, with 81.3% (13/16) being benign and 18.8% (3/16) malignant. These findings suggest a separation between low- and high-risk lesions, while the intermediate category remains heterogeneous. Overall, the observed distribution is consistent with clinical expectations, where the majority of IPMN lesions exhibit low to intermediate malignant potential.

#### 3.3.2. CT

Among the 97 CT examinations, IPMN was identified in 52.6% of cases. Application of the TDS resulted in classification as worth watching in 73.2%, worrisome in 25.9%, and high risk in 1.0% of examinations. As shown in Table 5, concordance between MRI- and CT-based TDS classification was observed in 75% of cases (39/52), with a moderate agreement (κ = 0.49, *p* < 0.001).

Lesions classified as worth watching on MRI were also classified as worth watching on CT in 90% of cases (27/30), while 10% (3/30) were upgraded to the worrisome category. For lesions classified as worrisome on MRI, 60% (12/20) remained worrisome on CT, whereas 40% (8/20) were downgraded to worth watching. Notably, lesions classified as high risk on MRI were consistently categorized as worrisome on CT (100%, 2/2), with no cases classified as high risk on CT. Overall, CT tended to classify lesions into lower risk categories compared with MRI.

#### 3.3.3. Ultrasound

A definitive sonographic diagnosis was feasible in 82 of 100 examinations, reflecting the inherent modality-dependent limitations of ultrasound. Within these examinations, IPMN was diagnosed in 50%. TDS classification categorized 63.4% as worth watching, 30.5% as worrisome, and 6.1% as high risk. As shown in Table 6, concordance between MRI and ultrasound-based TDS classification was observed in 65.1% of cases (41/63), corresponding to fair agreement (κ = 0.26, *p* = 0.017). Lesions classified as worth watching on MRI were also classified as worth watching on ultrasound in 76.7% of cases (33/43), while 23.3% (10/43) were upgraded to the worrisome category. Among lesions classified as worrisome on MRI, 38.9% (7/18) remained worrisome on ultrasound, whereas 44.4% (8/18) were downgraded to worth watching and 16.7% (3/18) were upgraded to high risk. Notably, lesions classified as high risk on MRI showed heterogeneous classification on ultrasound, with 50% (1/2) categorized as worrisome and 50% (1/2) as high risk. Overall, ultrasound-based TDS classification showed greater variability compared with MRI.

### 3.4. Intersections Between Imaging Modalities and Reference Standards

Direct overlap between imaging modalities and histopathology was observed in 25 MRI, 29 CT, and 15 US examinations. Given the limited overlap for certain modalities, MRI was used as the reference modality for intermodality comparisons. Using this approach, 52 CT examinations and 63 US examinations could be directly compared with MRI-based TDS classification.

### 3.5. Agreement Between MRI-Based TDS and Histopathology

Comparison of MRI-based TDS classification with histopathological malignancy classification showed concordant classification for lesions classified as worth watching and high risk. Within the worrisome category, the majority of lesions were histopathologically benign, while a smaller proportion represented malignant disease, indicating a tendency toward conservative overestimation in this intermediate-risk group.

### 3.6. Intermodality Agreement of the TDS

Intermodality comparison demonstrated substantial agreement between MRI- and CT-based TDS classifications and lower agreement between MRI and ultrasound. MRI provided the most consistent risk stratification across modalities. However, intermodality comparisons should be interpreted cautiously given the retrospective design, incomplete modality overlap, and modality-specific differences in imaging sensitivity.

### 3.7. Diagnostic Performance (Histopathological Subset)

In the histopathological IPMN subset (n = 25), diagnostic performance metrics for the detection of histopathologically confirmed malignant IPMN are summarized in Table 7. MRI demonstrated a sensitivity of 94.4% and a specificity of 57.1%. The positive predictive value (PPV) was 85.0%, and the negative predictive value (NPV) was 57.1%.

The Tübingen Dignity Score demonstrated a sensitivity of 40.0% and a specificity of 100%. The PPV was 100%, and the NPV was 87.0%. Fukuoka classification demonstrated a sensitivity of 100% and a specificity of 91.3%, with a PPV of 50.0% and an NPV of 100%.

These findings demonstrate distinct diagnostic profiles among the evaluated approaches. MRI and the Fukuoka criteria showed high sensitivity for the detection of malignant IPMN, whereas the TDS demonstrated maximal specificity within this cohort (100%, 95% CI: 83.2–100). However, this estimate should be interpreted cautiously, given the limited sample size and the resulting statistical uncertainty.

Table 7 summarizes the diagnostic performance of MRI, TDS, and the Fukuoka criteria in the histopathological subset. The comparison highlights distinct and complementary diagnostic profiles across the three approaches.

## 4. Discussion

In this retrospective single-center study, we evaluated the Tübingen Dignity Score (TDS) as a structured, imaging-based tool for risk stratification of intraductal papillary mucinous neoplasms (IPMNs). The principal finding of this work is an observed concordance between MRI-based TDS classification and histopathological findings in this cohort, particularly at the extremes of the risk spectrum, which should be interpreted with caution, given the limited number of malignant cases, especially in lesions categorized as worth watching and high risk. These findings suggest the potential applicability of TDS in scenarios where management decisions are most critical and are consistent with prior imaging-based risk stratification approaches [3,26,27].

### 4.1. Interpretation of the Principal Findings

Accurate differentiation between low-risk and high-risk IPMNs remains a central challenge in clinical practice. Within this cohort, the TDS demonstrated concordant classification of low-risk and high-risk lesions when compared with histopathology, although the small sample size precludes generalization of this finding. This observation aligns with previous studies demonstrating that imaging-based models perform best at the extremes of the risk spectrum [26,27,28,29].

The present study should be regarded as a proof-of-concept investigation. The proposed scoring approach was developed and evaluated within a retrospective single-center cohort and should therefore be considered exploratory and hypothesis-generating. Consequently, the observed performance characteristics require cautious interpretation and should not be viewed as definitive evidence of clinical utility until confirmed in independent external cohorts.

In contrast, the worrisome category showed a degree of overestimation, with approximately one-fifth of lesions proving malignant on histopathology. This reflects the well-documented biological heterogeneity of intermediate-risk IPMNs [5,17,30]. Importantly, cautious overestimation in this subgroup may be clinically preferable to underestimation, as it favors intensified surveillance rather than missed malignant transformation. Large surgical series have shown that up to 30–40% of IPMNs resected due to worrisome features harbor only low-grade dysplasia, highlighting the ongoing issue of overtreatment [31,32]. Conversely, population-based and longitudinal surveillance studies indicate that the majority of incidentally detected pancreatic cystic lesions remain indolent over long-term follow-up [33,34]. These data emphasize the need for structured tools that allow cumulative risk assessment beyond binary guideline criteria.

### 4.2. Direct Comparison with Fukuoka Criteria

A key finding of this study is the distinct diagnostic profile observed when comparing TDS with the Fukuoka criteria. In the histopathological subset, the Fukuoka classification demonstrated high sensitivity (100%) and high specificity (91.3%), with no malignant cases missed. However, the relatively low positive predictive value (50.0%) indicates that a substantial proportion of lesions classified as high risk did not harbor malignancy. This observation is consistent with previous reports demonstrating that Fukuoka criteria prioritize sensitivity to minimize the risk of missed malignancy, at the cost of potential overclassification and overtreatment [5,15,23]. In contrast, the TDS demonstrated high specificity (100%) and PPV (100%) in this cohort, indicating a strong ability to correctly identify high-risk lesions. However, this was accompanied by reduced sensitivity (40.0%), suggesting that some malignant lesions may not be captured by the score. These findings highlight a fundamental trade-off between sensitivity and specificity. While the Fukuoka criteria provide a safety-oriented approach suitable for screening and initial risk assessment, the TDS offers a more restrictive classification that may help reduce false-positive findings and unnecessary surgical interventions. Importantly, the inclusion of postoperative non-IPMN diagnoses within the surgically treated cohort reflects the real-world preoperative diagnostic challenge of differentiating suspected IPMN from alternative pancreatic cystic or malignant lesions during imaging-based risk assessment.

An important methodological aspect that must be considered when interpreting the comparative analysis between the TDS and the Fukuoka criteria is that the two approaches are not fully independent. Both systems are based on largely overlapping imaging features, including main pancreatic duct dilatation, mural nodules, cyst size, and wall characteristics, which are established markers of malignancy risk in IPMN. In particular, the Fukuoka criteria apply a rule-based framework designed to maximize sensitivity by capturing a broad range of potentially malignant lesions, whereas the TDS integrates the same or closely related imaging parameters into a weighted scoring system that emphasizes specificity. This lack of full independence is important to acknowledge in order to avoid overinterpreting the complementary nature of the two approaches. Instead, the findings should be understood as demonstrating how different structuring and weighting of similar imaging features can lead to varying balances between sensitivity and specificity within the same diagnostic domain.

Therefore, the observed differences should be interpreted as reflecting complementary diagnostic emphases rather than fully independent effects. Taken together, these findings support the use of the TDS as a complementary component within a multimodal diagnostic strategy, particularly in situations where clinical decision-making remains uncertain after initial risk stratification.

### 4.3. Integration of TDS into Clinical Workflow

Based on the complementary diagnostic profiles observed in this study, the TDS should not be considered a replacement for established guideline-based approaches but rather as an adjunctive tool. A stepwise diagnostic strategy may be most appropriate, in which MRI and guideline-based classification (e.g., Fukuoka criteria) serve as initial screening tools, followed by application of the TDS to refine risk stratification, particularly in patients with intermediate-risk features. Such multimodal and sequential approaches are increasingly supported by current guidelines and reviews, which emphasize individualized risk assessment by combining imaging, clinical, and, where available, molecular data [3,6,34,35]. In this context, the TDS may support more transparent risk stratification in multidisciplinary decision-making. Initial risk stratification should be performed using established criteria such as the Fukuoka guidelines or clinical judgment, which are designed to prioritize sensitivity and ensure that malignant lesions are not missed. Within this framework, the TDS may serve as a complementary tool in patients who are already considered for surgical evaluation. In particular, its potential value lies in clinical scenarios where high-risk features are identified based on Fukuoka criteria, but the overall clinical picture remains inconclusive, and the decision for surgical intervention is uncertain. In such cases, the TDS may provide additional specificity and support a more nuanced risk assessment. For example, a low TDS in patients classified as high risk according to Fukuoka criteria may suggest a lower likelihood of malignancy and could support a more conservative approach, such as intensified surveillance or additional diagnostic evaluation (e.g., EUS or biomarker analysis), rather than immediate surgical intervention. Conversely, a high TDS in the same setting may strengthen the indication for surgery by providing converging evidence of malignancy risk. Importantly, this proposed integration should be interpreted within the limitations of the present study. Given the limited number of histopathologically confirmed malignant cases, the role of the TDS in clinical decision-making should be considered exploratory and hypothesis-generating. Prospective validation in larger and more diverse cohorts is required before firm clinical recommendations can be established.

### 4.4. Comparison with Guideline-Based and Imaging-Based Models

International consensus and European guidelines provide a standardized framework for IPMN management by defining high-risk stigmata and worrisome features [3,4]. While these guidelines have improved consistency of care, several studies have demonstrated limited specificity, particularly in intermediate-risk lesions [31,36]. To address these limitations, multivariable nomograms and imaging-based risk models have been proposed. Composite imaging models and radiomics approaches have shown improved discrimination for malignant IPMNs compared with guideline criteria alone [13,37]. Similarly, structured risk stratification tools have been associated with enhanced clinical decision-making [35,38]. The TDS is conceptually aligned with these approaches by integrating multiple guideline-derived imaging features into a weighted and transparent scoring system. In contrast to radiomics-based or machine learning-driven models, which often require specialized software and extensive external validation [35,39], the TDS relies exclusively on routinely assessed imaging parameters. While this supports clinical applicability, it may limit the ability to capture more complex imaging patterns compared to advanced computational models. In light of the findings of this study, the TDS should be interpreted as a complementary approach rather than a replacement for established criteria. Its potential role lies in contributing to a more structured and transparent assessment within existing diagnostic frameworks.

### 4.5. Imaging Modality-Specific Performance

MRI was selected as the reference modality given its high sensitivity for detecting ductal communication, mural nodules, and subtle changes in the main pancreatic duct [33,40]. Multiple comparative studies have consistently demonstrated the diagnostic superiority of MRI, particularly when MRCP is included [15]. The substantial agreement observed between MRI- and CT-based TDS classifications supports the use of CT as an alternative modality when MRI is unavailable, in line with previous comparative imaging studies [30,40]. CT remains widely used due to its availability and high spatial resolution, particularly in preoperative settings. The relatively high number of CT examinations observed in our cohort reflects routine clinical practice, where pancreatic cystic lesions are frequently detected incidentally during abdominal CT examinations performed for unrelated clinical indications or at referring institutions. Consequently, CT often represents the initial imaging modality, with dedicated MRI subsequently performed for detailed lesion characterization and risk stratification.

Differences between MRI- and CT-based TDS classifications likely reflect modality-specific visualization and interpretation of imaging features relevant for risk stratification. Upgrading from “worth watching” to “worrisome” on CT may result from differing assessments of cyst size, main pancreatic duct dilatation, wall thickening, or suspected mural nodules. Conversely, downgrading may occur when findings considered suspicious on MRI are less conspicuous on CT or are interpreted differently because of variations in soft-tissue contrast and image resolution. These observations highlight inherent differences between imaging modalities and further support the role of MRI as the preferred modality for detailed characterization of IPMN.

Ultrasound showed lower agreement with MRI-based TDS, which is consistent with known limitations related to operator dependency and reduced sensitivity for small or deeply located pancreatic lesions [31,41]. Nevertheless, ultrasound-based TDS provided clinically relevant information in a subset of patients, supporting its role in follow-up examinations or in patients with contraindications to cross-sectional imaging.

### 4.6. Clinical Implications

From a clinical perspective, the TDS may support clinical decision-making regarding surgical versus conservative management and help balance the competing risks of overtreatment and delayed cancer detection. Longitudinal surveillance studies have demonstrated that main pancreatic duct dilatation and dynamic changes over time are among the strongest predictors of malignant transformation [32,33]. The weighted integration of these parameters within the TDS reflects their relative prognostic importance. Recent meta-analyses and clinical guidelines increasingly advocate individualized risk assessment rather than rigid size-based thresholds [34,42]. In this context, the TDS should be viewed as a complementary tool that enhances guideline-based decision-making rather than replacing established recommendations. Future integration of the TDS with molecular cyst fluid biomarkers or radiogenomic approaches may further improve predictive accuracy [35,43]. Future prospective multicenter studies are required to externally validate the proposed scoring approach across different patient populations, institutions, and imaging protocols. In addition, assessment of interobserver agreement will be essential to determine the reproducibility and robustness of score-based risk stratification in routine clinical practice.

### 4.7. Strengths and Limitations

The strengths of this study include the multimodal imaging approach, the use of a structured and reproducible scoring system, and the direct comparison with histopathology as the reference standard. However, several limitations must be acknowledged. The retrospective single-center design introduces potential selection bias and may limit generalizability. The number of cases with available histopathological confirmation was relatively small, particularly with regard to malignant lesions, which affects the robustness of sensitivity and predictive value estimates. Furthermore, interobserver variability was not assessed and may influence the reproducibility of both TDS and guideline-based classifications. The analysis was cross-sectional, and longitudinal changes in imaging features were not evaluated. Furthermore, differentiation between mural nodules and enhancing mural nodules was not consistently available in retrospective imaging documentation across the study period. Finally, the application of Fukuoka criteria was based on retrospective imaging data and may be influenced by incomplete feature documentation. Future prospective multicenter studies are required to validate these findings and to further define the role of the TDS within established diagnostic algorithms. In addition, diagnostic performance was calculated in the IPMN-positive histopathological subset, which may introduce selection bias and limit the generalizability of performance estimates.

An additional limitation relates to potential interobserver variability in the assessment of imaging features incorporated into the TDS. Several score components, including mural nodules, wall thickening, and subtle pancreatic duct abnormalities, may be subject to variability between radiologists and imaging centers. Because imaging evaluations were based on routine clinical reports and retrospective image review, formal assessment of interobserver agreement was beyond the scope of the present study. Consequently, the reproducibility of TDS classification remains uncertain. Future prospective studies should include independent blinded image review and formal interobserver analyses to determine the robustness and reproducibility of the scoring approach across different observers and institutions.

## 5. Conclusions

In conclusion, the Tübingen Dignity Score represents a structured imaging-based approach to risk stratification in IPMN. In this cohort, it showed a specificity-oriented diagnostic profile that may complement existing guideline-based strategies. Its potential clinical value lies in supporting decision-making in selected cases where established criteria yield inconclusive or conflicting results. However, given the limited number of malignant cases and the exploratory nature of this analysis, no definitive conclusions regarding its clinical utility can be drawn. Further external prospective multicenter validation in larger cohorts is required.

## Figures and Tables

**Table 1:** Point distribution of the Tübingen Dignity Score (TDS).

Point Distribution for Calculating the Tübingen Dignity Score (TDS)		
Findings	Scores	
Cyst < 10 mm	1 point	1. “Worth watching” (0–3 points)
Cyst 10–19.9 mm	2 points	
Cyst 20–29.9 mm	3 points	
Cyst > 30 mm	4 points	2. “worrisome” (4–24 points)
Mural nodules < 5 mm	4 points	
Thickened/perfused cyst walls	4 points	
Main pancreatic duct 5–9 mm	4 points	
Abrupt leaps in caliber in the main pancreatic duct	4 points	
Lymph node augmentation	4 points	
Mural nodules ≥ 5 mm	25 points	3. “High risk” (≥25 points)
Main pancreatic duct ≥ 10 mm	25 points	

Table 1 depicts the distribution of TDS risk categories across imaging findings.

**Table 2:** Overlap between available imaging modalities and histopathological reference examinations within the retrospective study cohort.

Number of Examinations with Intersections				
	MRI	CT	US	3D US
Pathology	25	29	15	0
MRI	---	52	63	11

Values indicate the number of patients who underwent both examinations listed in the corresponding row and column.

**Table 3:** Patients’ characteristics.

Examinations (n = 468)	Histology (n = 41)	MRI (n = 113)	CT (n = 97)	EUS (n = 49)	CEUS (n = 31)	US (n = 100)	Others * (n = 37)
Sex; n (%)							
Female	15 (36.6)	57 (50.4)	39 (40.2)	27 (55.1)	18 (58.1)	53 (53)	
Male	26 (63.4)	56 (49.6)	58 (59.8)	22 (44.9)	13 (41.9)	47 (47)	
Age (mean ± SD)							
Female	68.8 ± 9.1	67.3 ± 9.4	70.6 ± 8.3	65.6 ± 8.3	66.7 ± 9.3	67.2 ± 9.6	
Male	66.7 ± 6.5	65.3 ± 10.1	68.9 ± 8.9	65.4 ± 7.9	67.7 ± 9.5	71.0 ± 13.2	
Cyst size (mm) Mean ± SD	23.6 ± 17.5	18.7 ± 9.6	21.0 ± 12.6	16.6 ± 7.4	14.8 ± 11.0	15.8 ± 10.0	
Cyst size measured; n (%)	30 (73.2)	85 (75.2)	61 (62.9)	38 (77.6)	21 (66.7)	64 (64)	
Wall thickening							
yes	1	1	0	0	1	1	
no	29	84	61	38	20	63	
Intramural nodule							
yes	1	1	0	5	2	1	
no	29	84	61	33	19	63	
Pancreatic duct (PD)							
Duct width (mm) Mean ± SD	8.3 ± 4.8	6.0 ± 6.3	7.9 ± 5.9	5.2 ± 4.5	4.8 ± 1.3	5.6 ± 4.1	
PD dilatation n (%)	15 (36.6)	65 (57.5)	26 (26.8)	35 (71.4)	12 (38.7)	54 (54)	
5–9.9 mm; n (%)	6 (40)	3 (4.6)	1 (3.8)	3 (8.6)	0	4 (7.4)	
≥10 mm; n (%)	4 (26.7)	13 (20)	4 (15.4)	1 (2.9)	3 (25)	9 (16.7)	
No; n (%)	5 (33.3)	49 (75.4)	21 (80.8)	31 (88.6)	9 (75)	41 (75.9)	
IPMN positive n (%)	25 (61)	91 (82)	51 (52.6)	24 (49)	20 (64.5)	41 (41)	
BD-IPMN	19 (76)	57 (62.6)	10 (19.6)	12 (50)	8 (40)	15 (36.6)	
MD-IPMN	1 (4)	5 (5.5)	5 (9.8)	2 (8.3)	2 (10)	2 (4.9)	
Mixed	5 (20)	29 (31.9)	1 (2.0)	3 (12.5)	0	24 (58.5)	
Not classified	0	0	35 (68.6)	7 (29.2)	10 (50)	0	
IPMN negative n (%)	16 (39)	16 (17.6)	46 (47.4)	24 (49)	9 (29)	41 (41)	
IPMN detected (%)	100	94.4	43.9	51.6	73.9	52.0	

* Others: 3D-US, ERCP, FNA.

**Table 4:** TDS in MRI.

Comparison of the Score in MRI with the Histopathology.			
		Histopathology	
		Benign	Malignant
MRI Score	“Worth watching”	100% (7/7)	0
	“Worrisome”	81.3% (13/16)	18.8% (3/16)
	“High risk”	0	100% (2/2)

**Table 5:** Comparison of the TDS in MRI and CT.

Comparison of the Score in MRI and CT (n = 52).				
		Score CT		
		“Worth Watching”	“Worrisome”	“High Risk”
**Score MRI**	“Worth watching”	90% (27/30)	10% (3/30)	0
	“Worrisome”	40% (8/20)	60% (12/20)	0
	“High risk”	0% (0/2)	100% (2/2)	0

Concordance 75% (39/52), κ = 0.49, *p* < 0.001.

**Table 6:** Comparison of the score in MRI and ultrasound.

Comparison of the Score in MRI and Ultrasound (n = 63).				
		Score Ultrasound		
		“Worth Watching”	“Worrisome”	“High Risk”
**Score MRI**	“Worth watching”	76.7% (33/43)	23.3% (10/43)	0
	“Worrisome”	44.4% (8/18)	38.9% (7/18)	16.7% (3/18)
	“High risk”	0	50% (1/2)	50% (1/2)

Concordance 65.1% (41/63), κ = 0.26, *p* = 0.017.

**Table 7:** Diagnostic performance of MRI assessment, Fukuoka classification, and the Tübingen Dignity Score (TDS) for detection of histopathologically confirmed malignant IPMN within the histopathological IPMN subset (n = 25).

Metric	MRI	TDS	Fukuoka
Sensitivity	94.4% (95% CI: 72.7–99.9)	40.0% (95% CI: 5.3–85.3)	100% (95% CI: 15.8–100)
Specificity	57.1% (95% CI: 18.4–90.1)	100% (95% CI: 83.2–100)	91.3% (95% CI: 72.0–98.9)
PPV	85.0% (95% CI: 62.1–96.8)	100% (95% CI: 15.8–100)	50.0% (95% CI: 6.8–93.2)
NPV	80.0% (95% CI: 28.4–99.5)	87.0% (95% CI: 66.4–97.2)	100% (95% CI: 83.9–100)

Diagnostic performance metrics are presented with 95% confidence intervals (Clopper–Pearson). Values are based on the histopathologically confirmed IPMN subset (n = 25) derived from 41 patients who underwent surgical resection. Sensitivity, specificity, positive predictive value (PPV), and negative predictive value (NPV) refer to the detection of histopathologically confirmed malignant IPMN lesions within the histopathological IPMN subset.

## Data Availability

Data supporting the reported results are available from the corresponding author upon reasonable request.

## Abbreviations

The following abbreviations are used in this manuscript:

3D-US	three-dimensional ultrasound
BD-IPMN	Branch Duct Intraductal Papillary Mucinous Neoplasm
CA19-9	Carbohydrate Antigen 19-9
CEA	Carcinoembryonic Antigen
CEUS	contrast-enhanced ultrasound
CT	Computed Tomography
ERCP	Endoscopic retrograde cholangiopancreatography
FNA	fine-needle aspiration
IPMN	Intraductal Papillary Mucinous Neoplasm
MD-IPMN	Main Duct Intraductal Papillary Mucinous Neoplasm
MPD	main pancreatic duct
MRI	Magnetic Resonance Imaging
PDAC	pancreatic ductal adenocarcinoma
TDS	Tübingen Dignity Score
US	Ultrasound
